# What the papers say

**DOI:** 10.1093/jhps/hnw042

**Published:** 2016-11-10

**Authors:** Ajay Malviya

The Journal of Hip Preservation Surgery (JHPS) is not the only place where work in the field of hip preservation may be published. Although our aim is to offer the best of the best, we continue to be fascinated by work that finds its way into journals other than our own. There is much to learn from it so *JHPS* has selected six recent and topical articles for those who seek a brief summary of what is taking place in our ever-fascinating world of hip preservation. What you see here are the mildly edited abstracts of the original articles, to give them what *JHPS* hopes is a more readable feel. If you are pushed for time, what follows should take you no more than 10 min to read. So here goes …

## DOES THE DGEMRIC INDEX CHANGE AFTER SURGICAL TREATMENT OF FEMOROACETABULAR IMPINGEMENT?

Delayed gadolinium-enhanced MRI of cartilage (dGEMRIC) allows an objective, non-invasive and longitudinal quantification of biochemical cartilage properties. Although dGEMRIC has been used to monitor the course of cartilage degeneration after periacetabular osteotomy (PAO) for correction of hip dysplasia, such longitudinal data are currently lacking for femoroacetabular impingement (FAI).

Swiss researchers^1^ have conducted a prospective comparative, non-randomized, longitudinal study to answer these questions: (1) How does the mean acetabular and femoral dGEMRIC index change after surgery for FAI at 1-year follow-up compared with a similar group of patients with FAI treated without surgery? (2) Does the regional distribution of the acetabular and femoral dGEMRIC index change for the two groups over time? (3) Is there a correlation between the baseline dGEMRIC index and the change of patient-reported outcome measures (PROMs) at 1-year follow-up? (4) Among those treated surgically, can dGEMRIC indices distinguish between intact and degenerated cartilage?

At the time of enrolment, the patients’ decision whether to undergo surgery or choose non-operative treatment was not made yet. Thirty-nine patients (40 hips) who underwent either joint-preserving surgery for FAI (20 hips) or non-operative treatment (20 hips) were included. The two groups did not differ regarding Tönnis osteoarthritis score, preoperative PROMs or baseline dGEMRIC indices. There were more women (60% versus 30%, *P* = 0.003) in the non-operative group and patients were older (36 versus 30 years, *P* = 0.026) and had lower alpha angles (65° versus 73°, *P* = 0.022) compared with the operative group. The authors used a 3.0-T scanner and a three-dimensional dual flip-angle gradient-echo technique for the dGEMRIC technique for the baseline and the 1-year follow-up measurements. dGEMRIC indices of femoral and acetabular cartilage were measured separately on the initial and follow-up radial dGEMRIC reformats in direct comparison with morphologic radial images. Regions of interest were placed manually peripherally and centrally within the cartilage based on anatomic landmarks at the clockface positions. The WOMAC, the Hip disability and Osteoarthritis Outcome Score, and the modified Harris hip score were used as PROMs. Among those treated surgically, the intraoperative damage according to the Beck grading was recorded and compared with the baseline dGEMRIC indices.

Although both the operative and the non-operative groups experienced decreased dGEMRIC indices, the declines were more pronounced in the operative group (−96 versus −16 ms on the acetabular side and −96 versus −21 ms on the femoral side in the operative and non-operative groups, respectively; *P* < 0.001 for both). Patients undergoing hip arthroscopy and surgical hip dislocation experienced decreased dGEMRIC indices; the decline in femoral dGEMRIC indices was more pronounced in hips after surgical hip dislocation (−120 versus 61 ms, *P* = 0.002). In the operative group a decline in dGEMRIC indices was observed in 43 of 44 regions over time. In the non-operative group a decline in dGEMRIC indices was observed in 4 of 44 regions over time. The strongest correlation among patients treated surgically was found between the change in WOMAC and baseline dGEMRIC indices for the entire joint (*R* = 0.788, *P* < 0.001). Among those treated non-operatively, no correlation between baseline dGEMRIC indices and change in PROMs was found. In the posterosuperior quadrant, the dGEMRIC index was higher for patients with intact cartilage compared with hips with chondral lesions (592 versus 444 ms, *P* < 0.001).

The authors concluded that a decline in acetabular, femoral and regional dGEMRIC indices for the surgically treated group at 1-year follow-up despite an improvement in all PROMs. A similar but less pronounced decrease in the dGEMRIC index was observed in symptomatic patients without surgical treatment indicating continuous cartilage degeneration. Although treatment of FAI is intended to alter the forces acting across the hip by eliminating impingement, its effects on cartilage biology are not clear. dGEMRIC provides a non-invasive method of assessing these effects. Longer term studies will be needed to determine whether the matrix changes of the bradytrophic cartilage seen here are permanent or clinically important.

## IS INCREASED ACETABULAR CARTILAGE OR FOSSA SIZE ASSOCIATED WITH PINCER FEMOROACETABULAR IMPINGEMENT?

In a multi-centred research from Austria, Switzerland and USA the authors^2^ have tried to determine whether surgical treatment of pincer FAI should involve trimming of the prominent acetabular rim or reverse PAO that reorients the acetabulum. Rim trimming may decrease articular surface size to a critical threshold where increased joint contact forces lead to joint degeneration. Therefore, knowledge of how much acetabular articular cartilage is available for resection is important when evaluating between the two surgical options. In addition, it remains unclear whether the acetabulum rim in pincer FAI is a prominent rim because of increased cartilage size or increased fossa size.

Reformatted MR and CT data were used to establish linear length dimensions of the lunate cartilage and cotyloid fossa in normal, dysplastic and deep acetabula. The last 200 hips undergoing PAO, reverse PAO and surgical dislocation for acetabular rim trimming at one institution were included in the study. They compared MR images of symptomatic hips with acetabular dysplasia (20 hips), pincer FAI (29 hips) and CT scans of asymptomatic hips from patients who underwent CT scans for reasons other than hip pain (20 hips). These hips were chosen sequentially from the underlying pool of 200 potential subjects to identify the first 10 male and the first 10 female hips in each group that met inclusion criteria. As a result of low numbers all hips that had undergone reverse PAO and met inclusion criteria were included. Cartilage width was measured medially from the cotyloid fossa to the lateral labrochondral junction. Cotyloid fossa linear height was measured from superior to inferior and cotyloid fossa width was measured from anterior to posterior. Superior lunate cartilage width (SLCW) and cotyloid fossa height (CFH) were measured on MR and CT oblique coronal reformats; anterior lunate cartilage width (ALCW), posterior lunate cartilage width (PLCW) and cotyloid fossa width (CFW) were measured on MR and CT oblique axial reformats. Cohorts were compared using multivariate analysis of variance with Bonferroni’s adjustment for multiple comparisons.

Compared with control acetabula, dysplastic acetabula had smaller SLCW (2.08 versus 2.63 mm, mean difference = −0.55 mm; *P* < 0.01), ALCW (1.20 versus 1.64 mm, mean difference = −0.44 mm; *P* = 0.00), CFH (2.84 versus 3.42 mm, mean difference = −0.59 mm; *P* < 0.01), and CFW (1.98 versus 2.77 mm, mean difference = −0.80 mm; *P* < 0.0001). Based on the results, we identified two subtypes of deep acetabula. Compared with controls, deep subtype 1 had normal CFH and CFW but increased ALCW (2.09 versus 1.64 mm; *P* < 0.001) and PLCW (2.32 versus 2.00 mm; *P* = 0.04). Compared with controls, deep subtype 2 had increased CFH (4.37 versus 3.42 mm; *P* < 0.01) and CFW (2.76 versus 2.77 mm; *P* = 1.0) but smaller SCLW (2.12 versus 2.63 mm; *P* < 0.01).

The authors concluded that deep acetabula have two distinct morphologies: subtype one with increased anterior and posterior cartilage lengths and subtype two with a larger fossa in height and width and smaller superior cartilage length. In patients with deep subtype one hips that have increased anterior and posterior cartilage widths, rim trimming to create an articular surface of normal size may be reasonable. However, for patients with deep subtype two hips that have large fossas but do not have increased cartilage widths, we propose that a reverse PAO that reorients yet preserves the size of the articular surface may be more promising. However, these theories will need to be validated in well-controlled clinical studies.

## DO RADIOGRAPHIC PARAMETERS OF DYSPLASIA IMPROVE TO NORMAL RANGES AFTER BERNESE PAO?

The goal of PAO is to improve the insufficient coverage of the femoral head and achieve joint stability without creating secondary femoroacetabular impingement. However, the complex tri-dimensional morphology of the dysplastic acetabulum presents a challenge to restoration of normal radiographic parameters. Accurate acetabular correction is important to achieve long-term function and pain improvement. There are limited data about the proportion of patients who have normal radiographic parameters restored after PAO and the factors associated with under- and overcorrection.

Novais *et al*.^3^ aimed to determine the following (1) What is the proportion of patients undergoing PAO in which the acetabular correction as assessed by the lateral centre-edge angle (LCEA), anterior centre-edge angle (ACEA), acetabular inclination (AI) and extrusion index (EI) is within defined target ranges? (2) What patient and preoperative factors are associated with undercorrection of the acetabulum as defined by a LCEA <22°, a factor that has been reported to be associated with PAO failure at 10-year follow-up?

Between January 2007 and December 2011, 132 PAOs were performed in 116 patients for treatment of symptomatic acetabular dysplasia. One patient with Legg-Calvé-Perthes disease, one with multiple osteochondromatosis, and two with concomitant femoral osteotomy were excluded. A total of 128 hips (112 patients) were included. The hip cohort was 76% (97 of 128) female and the mean age at surgery was 28.5 years (SD 8.7 years). Correction of LCEA between 25° and 40°, ACEA between 18° and 38°, Tönnis angle between 0° and 10° and EI ≤20% were defined as adequate based on normative values. Values lower than the established parameters were considered undercorrection for the LCEA and ACEA and those higher than the established values were considered overcorrection. Because postoperative LCEA <22° has been previously associated with PAO failure at a minimum of 10-year follow-up, in this study the authors sought to measure whether demographic factors including age, gender, body mass index and severity of acetabular dysplasia assessed by preoperative LCEA, ACEA, AI and EI were associated with undercorrection. Postoperative radiographs were obtained at minimum of one month after surgery (mean, 7 months; range 1–44 months) and were measured by a professional research assistant and a hip reconstruction fellow not involved in the clinical care of the patients. No patient was lost to follow-up.

Of the 128 hips, the proportion of hips with radiographic parameters within the established range was 78% (100 hips) for the LCEA, 86% (110 hips) for the ACEA, 89% (114 hips) for the AI and 80% (102 hips) for the EI. For hips with an inadequate correction, the LCEA was more often undercorrected than overcorrected (20 versus 2%; 95% confidence interval [CI], 11–27%; *P* < 0.001), whereas the ACEA was more often overcorrected than undercorrected (11 versus 3%; 95% CI 1–15%; *P* = 0.03) After adjusting for age, sex, body mass index and preoperative radiographic parameters including ACEA, AI and EI, we found that the preoperative LCEA was the only independent factor associated with a postoperative LCEA <22° (odds ratio 0.92; 95% CI 0.87–0.97; *P* = 0.003), indicating that hips with lower preoperative LCEA were more likely to have a LCEA <22°. For each additional degree of preoperative LCEA, the odds of LCEA <22° were reduced by 15%.

Acetabular correction after PAO performed by two experienced surgeons was adequate for individual radiographic parameters in most but not all hips. Hips with more severe dysplasia preoperatively are at higher risk for undercorrection as assessed by the LCEA. This intuitive information may help surgeons performing PAO in severely dysplastic hips plan for possible combined procedures including a femoral osteotomy if PAO alone does not allow for adequate correction of femoral head coverage and a congruous concentric hip. Further studies are planned to determine whether the long-term hip function and pain in patients whose hips were corrected within these established parameters will be improved in comparison to those that were under- or overcorrected.

## WOULD INJECTABLE AUTOLOGOUS CHONDROCYTE HELP IN FULL THICKNESS ACETABULAR CARTILAGE DEFECTS?

Acetabular cartilage lesions are frequently seen in young patients with hip pain and have been identified as an important prognostic factor. Schroeder *et al.*^4^ from the Centre for musculoskeletal surgery, Berlin, Germany have evaluated the early outcome of patients with arthroscopic injectable autologous chondrocyte transplantations (ACT) for full thickness acetabular cartilage defects.

A two-step procedure ACT was performed in patients with full thickness acetabular cartilage defects measuring ≥2 cm. The patients were closely followed with clinical examination, pre- and postoperative scores until the latest available follow-up of 3, 6, 12 and 24 months. Twenty consecutive cases (4 female, 16 male, mean age 33 years) were included. No patients were lost at final follow-up. The average defect size was 5.05 (range 2–6) cm. The average follow-up was 12.05 (range 6–24) months.

Three months postoperatively the preoperative scores improved significantly from a mean mHHS of 63–81 points (*P* = 0.009), iHOT33 of 44–66% (*P* = 0.028) and subjective hip assessment (Subjective Hip Value, SHV) from 60 to 87% (*P* = 0.007). After 12 months the results improved significantly to a mean mHHS of 93 points (*P* = 0.017), an iHOT33 of 79% (*P* = 0.007) and an SHV of 82% (*P* = 0.048) compared with the preoperative scores.

The injectable matrix-associated ACT has been proposed as a reliable procedure, yielding promising early results with a significant increase of all scores evaluated in patients with full thickness acetabular cartilage defects.

## SHOULD HIP ARTHROSCOPY BE OFFERED TO PATIENTS OVER THE AGE OF 40?

In a multicentred collaboration^5^ between McMaster University, Canada and Steadman Philippon Research Institute, USA, a systematic review was performed to (1) report clinical outcomes, complication rates, and total hip arthroplasty (THA) conversion rates for patients age 40 or older who underwent hip arthroscopy, and (2) report any age-related predictors of outcome identified in the literature.

MEDLINE, EMBASE and PubMed were searched for relevant studies and pertinent data were abstracted from eligible studies. No meta-analysis was performed because of heterogeneity amongst studies.

Seventeen studies were included in this review comprising 16 327 patients, including 9 954 patients age 40 or older. All studies reported statistically significant improvements in outcomes after hip arthroscopy for femoral osteochondroplasty, labral repair or unspecified indications. In patients 40 or older who underwent labral debridement, these improvements were not clinically significant. Obesity and osteoarthritic changes predicted poorer outcomes. Only one of three studies directly comparing the two groups found that patients 40 or older had a significantly less improvement in a standardized hip outcome score than patients under 40 after hip arthroscopy, but all found that patients 40 or older had significantly higher rates of THA conversion. The rate of conversion to THA was 18.1% for patients 40 or older, 23.1% for patients over 50 and 25.2% for patients over 60 with a mean of 25.0 months to THA.

The study concluded that femoral osteochondroplasty and labral repair resulted in clinically significant improvements in patients 40 or older in most research studies examined in this review, whereas labral debridement did not produce clinically significant improvements postoperatively in the same studies. In these studies, the rate of conversion to THA is higher than in patients under 40 and increases with each decade of life, with many individual studies showing a significant increase in the rate of THA conversion.

Hip arthroscopy may be suitable for some patients 40 or older, but patient selection is key and patients should be informed of the higher risk of conversion to THA.

## DOES SIMVASTATIN HAVE A ROLE IN TREATMENT OF AVASCULAR NECROSIS OF FEMORAL HEAD?

Yin *et al*.^6^ from Shandong, China, looked at multiple drilling combined with simvastatin versus multiple drilling alone for the treatment of avascular osteonecrosis of the femoral head at a 3-year follow-up. Simvastatin has been demonstrated to promote bone formation and reduce bone adsorption. The purpose of this study was to determine whether simvastatin enhanced the effect of multiple decompressions in preventing progression of ANFH and to identify independent risk factors associated with poor results.

The authors retrospectively analysed 58 hips in 36 patients, with a follow-up of 36 months. Twenty patients (32 hips) underwent multiple drilling combined with simvastatin treatment (SIM group); 16 patients (26 hips) underwent multiple drilling alone (MD group). Clinical failure was defined as a requirement for subsequent hip surgery or Harris Hip Score <75. New occurrence of collapse or increased collapse >2 mm on plain radiographs was defined as radiological failure.

Successful clinical results were achieved in 27 of 32 hips (84%) in the SIM group compared with 15 of 26 hips (58%) in the MD group (OR = 0.2, *P* = 0.032). Successful radiological results were achieved in 27 of 32 hips (84%) in the SIM group and in 16 of 26 hips (61.5%) in the MD group (*P* = 0.048). Body mass index, disease stage and location of lesion were independent prognostic factors for overall survival.

The study concluded that simvastatin could enhance the effects of multiple decompressions in preventing progression of ANFH and reducing the risk of femoral head collapse.

## CONFLICT OF INTEREST STATEMENT

None declared.
